# Genetic Perturbation of Pyruvate Dehydrogenase Kinase 1 Modulates Growth, Angiogenesis and Metabolic Pathways in Ovarian Cancer Xenografts

**DOI:** 10.3390/cells10020325

**Published:** 2021-02-05

**Authors:** Carolina Venturoli, Ilaria Piga, Matteo Curtarello, Martina Verza, Giovanni Esposito, Santina Venuto, Filippo Navaglia, Angela Grassi, Stefano Indraccolo

**Affiliations:** 1 Immunology and Molecular Oncology Unit, Veneto Institute of Oncology IOV—IRCCS, 35128 Padova, Italy; carolinaventuroli@live.it (C.V.); ilaria.piga1992@gmail.com (I.P.); matteo.curtarello@iov.veneto.it (M.C.); martina2.verza@gmail.com (M.V.); giovanni.esposito@iov.veneto.it (G.E.); angela.grassi@iov.veneto.it (A.G.); 2 Department of Surgery, Oncology and Gastroenterology, University of Padova, 35128 Padova, Italy; 3 Department of Biology, University of Padova, 35128 Padova, Italy; santina.venuto@studenti.unipd.it; 4 Department of Laboratory Medicine, University Hospital of Padova, 35128 Padua, Italy; filippo.navaglia@aopd.veneto.it

**Keywords:** ovarian cancer, PDK1, metabolism, glycolysis, angiogenesis

## Abstract

Pyruvate dehydrogenase kinase 1 (PDK1) blockade triggers are well characterized in vitro metabolic alterations in cancer cells, including reduced glycolysis and increased glucose oxidation. Here, by gene expression profiling and digital pathology-mediated quantification of in situ markers in tumors, we investigated effects of PDK1 silencing on growth, angiogenesis and metabolic features of tumor xenografts formed by highly glycolytic OC316 and OVCAR3 ovarian cancer cells. Notably, at variance with the moderate antiproliferative effects observed in vitro, we found a dramatic negative impact of PDK1 silencing on tumor growth. These findings were associated with reduced angiogenesis and increased necrosis in the OC316 and OVCAR3 tumor models, respectively. Analysis of viable tumor areas uncovered increased proliferation as well as increased apoptosis in PDK1-silenced OVCAR3 tumors. Moreover, RNA profiling disclosed increased glucose catabolic pathways—comprising both oxidative phosphorylation and glycolysis—in PDK1-silenced OVCAR3 tumors, in line with the high mitotic activity detected in the viable rim of these tumors. Altogether, our findings add new evidence in support of a link between tumor metabolism and angiogenesis and remark on the importance of investigating net effects of modulations of metabolic pathways in the context of the tumor microenvironment.

## 1. Introduction

Tumor cells commonly disclose higher rates of glycolysis than normal cells [1], a metabolic alteration also known as the Warburg effect, which represents the best-known metabolic hallmark of cancer [2]. Notably, PET clinical studies indicate that highly glycolytic tumors of various histologies—defined as tumors which show increased standardized uptake value (SUV) of 2-[(18)F] fluoro-2-deoxy-D-glucose and high total lesion glycolysis—have more aggressive behaviors [3,4,5,6]. Along the same line, Walenta et al. reported that lactate-rich tumor samples—measured by induced metabolic bioluminescence imaging (imBI)—have dismal prognoses in head and neck cancer and several additional cancer types (reviewed in [7,8]).

Hypothetically, as the Warburg effect is associated with cell proliferation both in normal and cancer cells [9], this link could possibly explain the accelerated rate of tumor growth associated with highly glycolytic tumors. However, the scenario is likely to be more complex as indirect protumor effects of glycolysis have also been described. It could be speculated that soluble factors released by highly glycolytic tumor cells, such as lactic acid or an acidic pH, modulate the stromal microenvironment, contributing to promote angiogenesis and making it more favorable to tumor growth [10]. Lactate can be picked up by stromal cells, such as fibroblasts and macrophages, and used to feed oxidative phosphorylation [11]. Moreover, in certain experimental models lactate appears to stabilize HIF1α in the stroma, leading to increased production of proangiogenic cytokines, including VEGF and bFGF [10]. Lactate influx can also stimulate CXCL-8 production in endothelial cells (ECs) [12].

We recently described a set of metabolism-related markers which can be used to investigate by immunohistochemistry (IHC) the metabolic make-up of tumors and by profiling patient-derived ovarian cancer xenografts (PDX) samples we observed that expression of MCT4, a monocarboxylate transporter associated with glycolysis [13], was negatively associated with survival of mice bearing intraperitoneal tumors [14]. In our study, we reported correlative data indicating that MCT4 expression was positively associated with proliferation and microvessel density but we had no direct evidence linking glycolysis to cell proliferation and angiogenesis in ovarian cancer models.

Pyruvate dehydrogenase kinase (PDK) is a canonical regulator of glycolysis and it has been at the center of significant basic and clinical research interests in the past decade as a pharmacologic target of dichloroacetate (DCA) [15]. Previous studies illustrated how DCA shifts tumor metabolism from glycolysis to glucose oxidation and the impressive effects of PDK blockade on growth of various types of experimental tumors [16]. It also appears that DCA impacts tumor angiogenesis by reverting the mitochondrial signals that lead to HIF1α activation in cancer [17].

Most information related to effects of PDK blockade stem from in vitro studies with DCA, which enable accurate measurements of metabolic effects on tumor cells. However, growth culture conditions might only mimic the complexity of the tumor microenvironment in part and there are examples showing that certain metabolic pathways activated by tumor cells in vitro are barely utilized in the context of solid tumors [18]. With this concern in mind, we aimed to investigate effects of Pyruvate dehydrogenase kinase 1 (PDK1) blockade in tumors. To this end, we silenced PDK1 by lentiviral vector delivery of shRNA and investigated by digital pathology and microarray analysis morphologic and transcriptomic changes caused by this modulation in ovarian cancer xenografts.

## 2. Materials and Methods

### 2.1. Cell Culture and Treatments

Two ovarian cancer cell lines (OC316 and OVCAR3), representative of highly glycolytic cells [19,20], were used in this study. OC316 cells were provided by S. Ferrini (IST, Genoa, Italy) and OVCAR3 cells were provided by S. Canevari (INT, Milan, Italy). OC316 and OVCAR3 were cultured in RPMI 1640 (Euroclone, Milan, Italy) supplemented with 10% fetal calf serum (FCS, Life Technologies, Paisley, UK), 1% HEPES (10mmol/L, Cambrex Bioscence, East Rutherford, NJ, USA), 1% Sodium Pyruvate (1 mmol/L), 1% L-glutamine (2 mmol/L), and 1% antibiotic–antimycotic mix 100× (Gibco–Thermo Fisher Scientific, Waltham, MA, USA). The human embryonic kidney epithelium cell line 293T was purchased from ATCC and cultured in Dulbecco modified Eagle medium (Euroclone), supplemented with 10% FCS, 10 mM HEPES and 1% of antibiotic–antimycotic mix (Thermo Fisher Scientific) and used to produce lentiviral vectors. Cultures were maintained at 37 °C in humidified 5% CO_2_/95% air atmosphere.

### 2.2. In Vivo Experiments

For tumor establishment, 8-week-old NOD/SCID mice were purchased from Charles River Laboratories (Wilmington, MA, USA) and housed in our specific pathogen-free animal facility, and were subcutaneously injected into both flanks with 0.3 × 10^6^ shRNA or shPDK1 cells mixed at +4 °C with liquid Matrigel (Becton Dickinson; Franklin Lakes, NJ, USA). Tumor volume (mm^3^) was measured by a caliper and calculated according to the following formula: L × l2 × 0.5, where L is the longest diameter, l is the shortest diameter, and 0.5 is a constant to calculate the volume of an ellipsoid. Once mice developed tumors, they were anaesthetized with isoflurane/oxygen and sacrificed by cervical dislocation. Procedures involving animals and their care were conformed to institutional guidelines that comply with national and international laws and policies (EEC Council Directive 86/609, OJ L 358, 12 December 1987) and were authorized by the Italian Ministry of Health (authorization no. 617/2016-PR).

### 2.3. Lentiviral Vector-Mediated PDK1 Gene Silencing

Lentiviral plasmids encoding shRNA-targeting human PDK1 gene and control shRNA were purchased from Sigma-Aldrich (St. Louis, MO, USA) and transfected in 293T cells to generate lentiviral vectors. Ovarian cancer cells expressing the different vectors were selected in puromycin-containing medium 1 ug/mL for 10–14 days prior to subsequent analysis.

### 2.4. Reverse Transcription-PCR (RT-PCR) and Quantitative PCR (qPCR)

Total RNA was isolated using TRIzol Reagent (Thermo Fisher Scientific) according to the manufacturer’s instructions. cDNA was synthesized from 1–1.5 μg of total RNA using the High Capacity RNA-to-cDNA kit (Thermo Fisher Scientific). For analysis of PDK1 silencing, real-time PCR was performed with SYBER Green dye and 7900HT Fast Real-Time PCR System (Thermo Fisher Scientific). Cycling conditions were 10 min at 95 °C, 40 cycle of 15 s at 95 °C and 1 min at 60 °C. Each sample was run in duplicate. For all genes evaluated, mRNA was normalized to ß2-microglobulin (B2M) mRNA by subtracting the cycle threshold (Ct) value of B2M mRNA from Ct value of the gene of interest (ΔCt). Fold difference (2^−ΔΔCt^) was calculated by subtracting the ΔCt (shPDK1 sample) to ΔCt (reference sample), to generate a ΔΔCt.

### 2.5. Lactate Measurements

Lactate concentrations in supernatants were determined on an automated analyzer (Cobas 8000, Roche, Basel, Switzerland). Values were normalized to protein number at the end of the incubation period.

### 2.6. Seahorse Analysis

Oxygen consumption rate (OCR) and extracellular acidification rate (ECAR) were assessed in real time with a XF24 Extracellular Flux Analyzer (Seahorse Biosciences, Billerica, MA, USA). Cells (2.5 × 10^4^/well) were plated in RPMI medium supplied with 10% FCS. The following day, cells were placed in a running DMEM medium (supplemented with 25 mmol/L D-glucose, 2 mmol/L glutamine, 1 mmol/L sodium pyruvate and without serum and bicarbonate) and preincubated for 30 min at 37 °C in atmospheric CO_2_ before starting metabolic measurements. At the end of the experiment, OCR and ECAR values were normalized for the protein content of each sample. Accurate titration with the uncoupler FCCP was performed for each cell type, to utilize the FCCP concentration (400 nmol/L) that maximally increases OCR without being toxic.

### 2.7. Proliferation Assay

The sulforhodamine (SRB) assay was used for testing differences in proliferation between silenced (shPDK1) and control (shRNA) cells based on the measurement of cellular protein content. After an incubation period, cell monolayers are fixed with 10% (*wt/vol*) trichloroacetic acid and stained for 30 min, after which the excess dye was removed by washing repeatedly with 1% (*vol/vol*) acetic acid. The protein-bound dye was dissolved in 10 mM Tris base solution for OD determination at 510 nm using a microplate reader.

### 2.8. PKH26 Staining

Cell division was evaluated using PKH26 staining (Sigma Aldrich). Labeling was performed with a 1:250 (*v*/*v*) PKH26 solution (Sigma-Aldrich). After 3 min of incubation, staining was blocked with 1% BSA. Cells were seeded in 6 wells at 1 × 10^5^/well and incubated for 24 h at 37 °C. Analysis was performed 24 h later with the FACS LRSRII Flow cytometry (BD Bioscience, Franklin Lakes, NJ, USA).

### 2.9. Immunoblotting Assay

Whole-cell lysates (5 × 10^5^ cells) were prepared in RIPA lysis buffer (Cell Signaling Technology, Denvers, MA, USA) containing a protease and a phosphatase inhibitor cocktail (Sigma Aldrich). Proteins were quantified using Quantum Protein Assay (EuroClone), and about 30 μg were denatured and loaded in a midi polyacrylamide gel (4–12%) (Thermo Fisher Scientific). Separated proteins were transferred for 2.5 h at 400 mA onto a nitrocellulose membrane (GE Health Care, Glattbrugg, Switzerland). Membranes were saturated overnight at 4 °C with Tris-buffered saline −0.1% Tween −5% milk and then incubated with primary antibody, according to the manufacturer’s instructions. Immunoprobing was performed using anti-PDK1 Rabbit mAb (clone C47H1, dilution 1:1000, from Cell Signaling) and anti-α-Tubulin Rabbit mAb (clone T5168, dilution 1:4000, from Sigma Aldrich), and it was followed by hybridization with horseradish peroxidase-conjugated anti-rabbit or anti-mouse Ab (Perkin Elmer, Waltham, MA, USA). The antigens were identified by luminescent visualization using Western Lightning Plus ECL reagents (Perkin Elmer), and signal intensity was detected using UVITEC Alliance Software (Cambridge, UK). Protein expression was assessed and normalized to tubulin (Sigma Aldrich) as housekeeping gene.

### 2.10. Histology and Immunohistochemistry

Three-micron-thick formalin-fixed paraffin-embedded (FFPE) tumor samples were stained either with hematoxylin and eosin and processed for IHC. In this case, IHC was performed with an automatic stainer (BOND III, Leica Microsystems, Wetzlar, Germany), and by using the following antibodies, according to the manufacturer’s instructions: anti-phospho-Histone H3 (pHH3) Rabbit polyclonal Ab (1:100 dilution, Cell Signaling Technology), anti-Caspase3 Rabbit (1:50 diluition, Cell Signaling Technology) and anti-mouse CD31 Rat mAb (clone SZ31, dilution 1:20, DIANOVA GmbH-Hamburg Germany).

### 2.11. Image Acquisition and Analysis

Tumor representation and quality of staining were initially evaluated by one experienced pathologist (GE). Images were digitally acquired at 20X magnification by the Aperio CS2 (Leica Biosystems, Wetzlar, Germany), and the evaluation of the IHC score was assessed through Scanscope Image Analysis software (ImageScope v12.4.0.708). On the basis of their localization, the different markers were analyzed by using the Aperio nuclear algorithm (pHH3, Caspase3), and the microvessels analysis v1 algorithm (CD31). The results provided by nuclear algorithm indicates the percentage of cells with different expressions of the nuclear marker classified as 3+ (highly positive), 2+ (intermediate positive), 1+ (low positive) and 0 (negative). To calculate pHH3 and Caspase 3 positive cells, only 3+ values were considered. Microvessels analysis (CD31) indicated both the area (MVA) and the density (MVD). Finally, digital quantification performed by means of the software was comparable with pathologist semiquantitative evaluation.

### 2.12. Microarray Expression Analysis

RNA quality and purity were assessed with the Agilent Bioanalyzer 2100 (Agilent Technologies; Waldbronn, Germany, Europe) and the eukaryote total RNA nanoassay (Agilent). For microarray expression experiments, only RNA of high quality was employed (RIN > 6). RNA samples that passed quality controls were diluted to 100 ng in a total volume of 3 μL DEPC-treated water. The GeneChip 3′ IVT PLUS Reagent Kit was used to prepare hybridization-ready targets of shRNA and shPDK1 OVCAR3 tumors. Following fragmentation, biotinylated cRNA was hybridized for 16 h at 45 °C to GeneChip™ PrimeView™ Human Gene Expression Arrays in an Affymetrix GeneChip hybridization oven 645. Affymetrix Fluidics Station 450 was used to stain and wash the chips. Arrays were then scanned on an Affymetrix GeneChip Scanner GCS3000 and the image (*.DAT) files were processed using the Affymetrix GeneChip Command Console (AGCC) software v.5.0 to generate cell intensity (*.CEL) files. Prior to transcriptional data analysis, a chip quality assessment was executed using the Affymetrix Expression Console software v.1.4 and for every array, all quality metrics were found within boundaries.

Bioinformatics analysis was carried out in the R statistical environment using Bioconductor packages [21] and customized code. Data were preprocessed using the RMA algorithm [22]. Differential expression analysis was performed using the limma package, by linear modelling, moderating the t-statistics by empirical Bayes shrinkage [23]. To correct for multiple testing, the Benjamini and Hochberg’s (BH) method was applied and genes with BH adjusted *p*-value < 0.01 were considered significant.

The functional significance of curated sets of genes was evaluated by Gene Set Enrichment Analysis (GSEA) [24]. Genes were ranked by moderated t-statistics and fast preranked GSEA, as implemented in [25], was run against the KEGG canonical pathways present in the “c2.cp.kegg” subcollection of the Molecular Signatures Database. Gene sets with a Benjamini–Hochberg adjusted *p*-value < 0.10 were considered significantly enriched.

Microarray data, together with the description of experiments and protocols, have been deposited in the ArrayExpress database (www.ebi.ac.uk/arrayexpress) under accession number E-MTAB-9877 (released on 31 December 2020).

### 2.13. Statistical Analysis

Results were expressed as mean value ± SD. Statistical comparisons between two samples were performed using the nonparametric Mann–Whitney test. Differences were considered statistically significant when *p* ≤ 0.05.

## 3. Results

### 3.1. PDK Silencing Modulates Glycolysis and In Vitro Growth of Ovarian Cancer Cells

**Figure 1:**
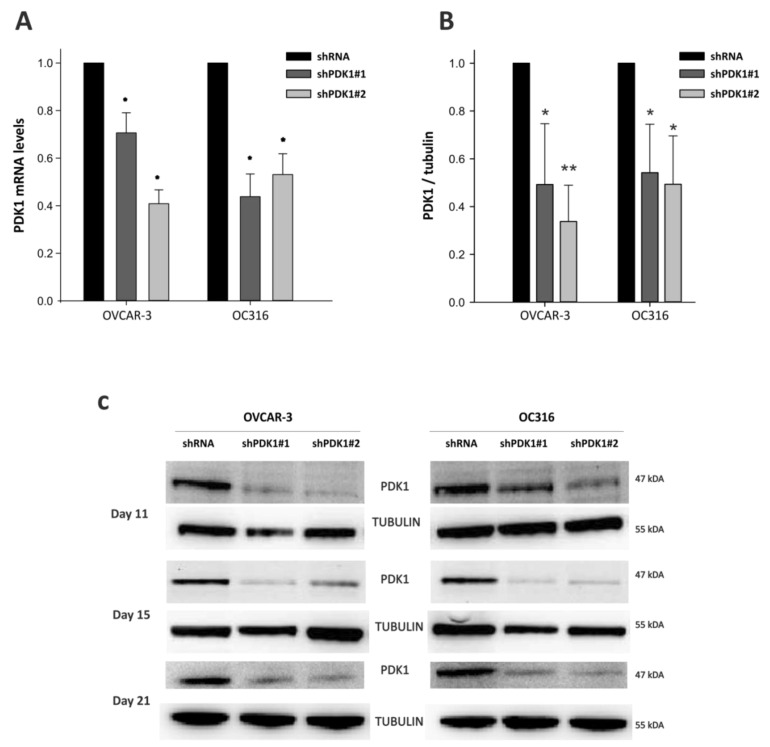
Decreased expression of Pyruvate dehydrogenase kinase 1 (PDK1) at mRNA and protein levels. OVCAR-3 and OC316 cell lines were transduced by lentiviral vectors encoding PDK1-targeting shRNA (shPDK1#1 and shPDK1#2). (**A**) PDK1 mRNA expression levels were analyzed by real-time PCR, * *p* < 0.05 Mann–Whitney. (**B**) PDK1 protein expression levels were quantified by Western blot analysis. * *p* < 0.05, ** *p* < 0.01, *t*-test. (**C**) PDK1 silencing was stable in both vectors for at least 21 days in vitro according to Western blot analysis. Quantification of protein levels was normalized to α-Tubulin. Data are mean ± SD values of two replicates normalized to protein content.

**Figure 2:**
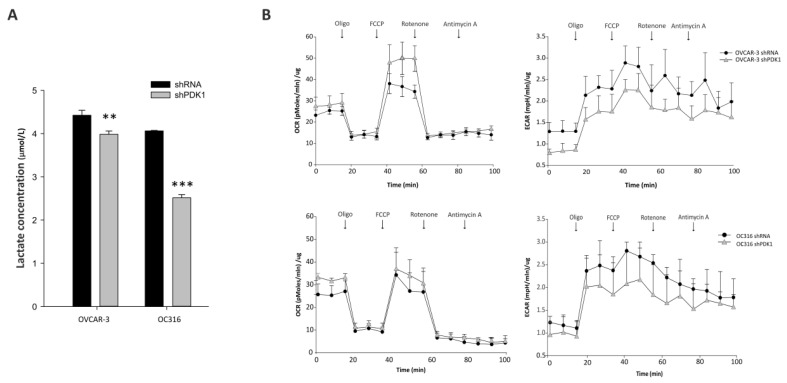
Effects of PDK1 silencing on glycolysis and cell metabolism. (**A**) Measurement of lactic acid production by PDK1-silenced and control cells, normalized by total protein content, ** *p* < 0.01, *** *p* < 0.001, *t* test. (**B**) Representative Oxygen consumption rate (OCR) and extracellular acidification rate (ECAR) traces obtained from shPDK1 and control cells. Serial additions of the ATP synthase inhibitor oligomycin, of the uncoupler FCCP, of the ETC complex I inhibitor rotenone, and of the respiratory complex III inhibitor antimycin A were carried out. Data are mean ± SD values of three replicates normalized to protein content.

**Figure 3:**
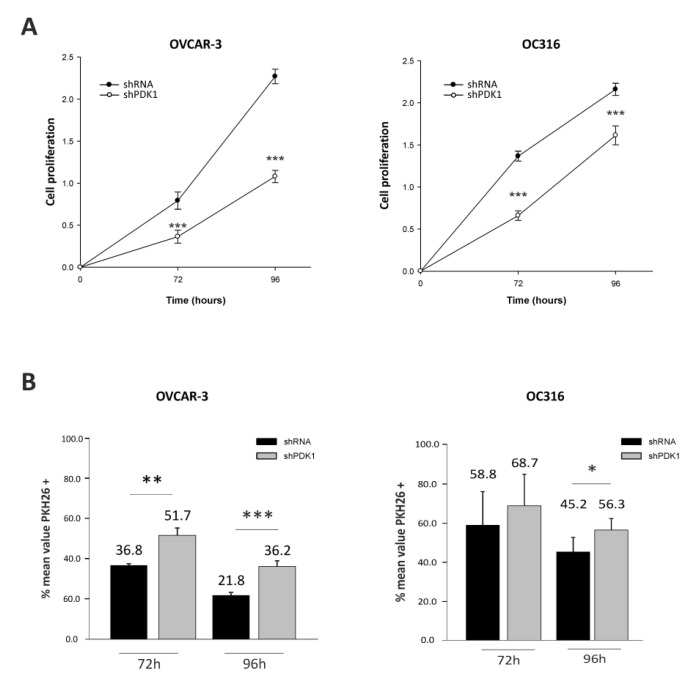
Effects of PDK1 silencing on cell proliferation. (**A**,**B**) Cell proliferation was reduced in PDK-silenced cells compared to control cells by sulforhodamine (SRB) assay and was confirmed by PKH26 staining. * *p* < 0.05, ** *p* < 0.01, *** *p* < 0.001, *t* test. Data are mean ± SD values of two replicates.

In initial experiments, PDK was silenced by lentiviral vector-mediated delivery of shRNA into OC316 and OVCAR-3 ovarian cancer cells. These cancer cell lines were used in previous studies and are prototypes of highly glycolytic tumor cells [19,20]. As shown in Figure 1A, substantial attenuation of PDK mRNA expression was obtained in both cancer cell lines by two different shRNA vectors targeting the human PDK1 gene. Reduced PDK expression was confirmed by WB analysis at the protein level using a PDK1-specific primary antibody (Figure 1B). Moreover, PDK silencing was stable for at least 21 days in vitro (Figure 1C). We next investigated effects of PDK silencing on glycolysis. Reduced concentrations of lactate were measured in supernatants from PDK-silenced OC316 and OVCAR3 cells compared with control cells (Figure 2A), with more marked reduction in the case of OC316 cells. We further investigated effects of PDK silencing on cell metabolism by using Seahorse analysis which showed a slight increase in oxygen consumption rate (OCR) and a decrease in extracellular acidification rate (ECAR) in shPDK1 cells compared to shRNA tumor cells (Figure 2B). Notably, these metabolic effects were accompanied by reduced cell proliferation, which was detected after 72 and 96 h from plating by the SRB assay (Figure 3A) and confirmed by PKH26 staining (Figure 3B), a fluorescent dye used to monitor cell division [26]. In conclusion, in vitro assays showed that stable PDK silencing in ovarian cancer cells can be achieved and it is accompanied by reduced glycolytic activity and cell proliferation.

### 3.2. Effects of PDK Silencing on Tumor Growth and Angiogenesis

**Figure 4:**
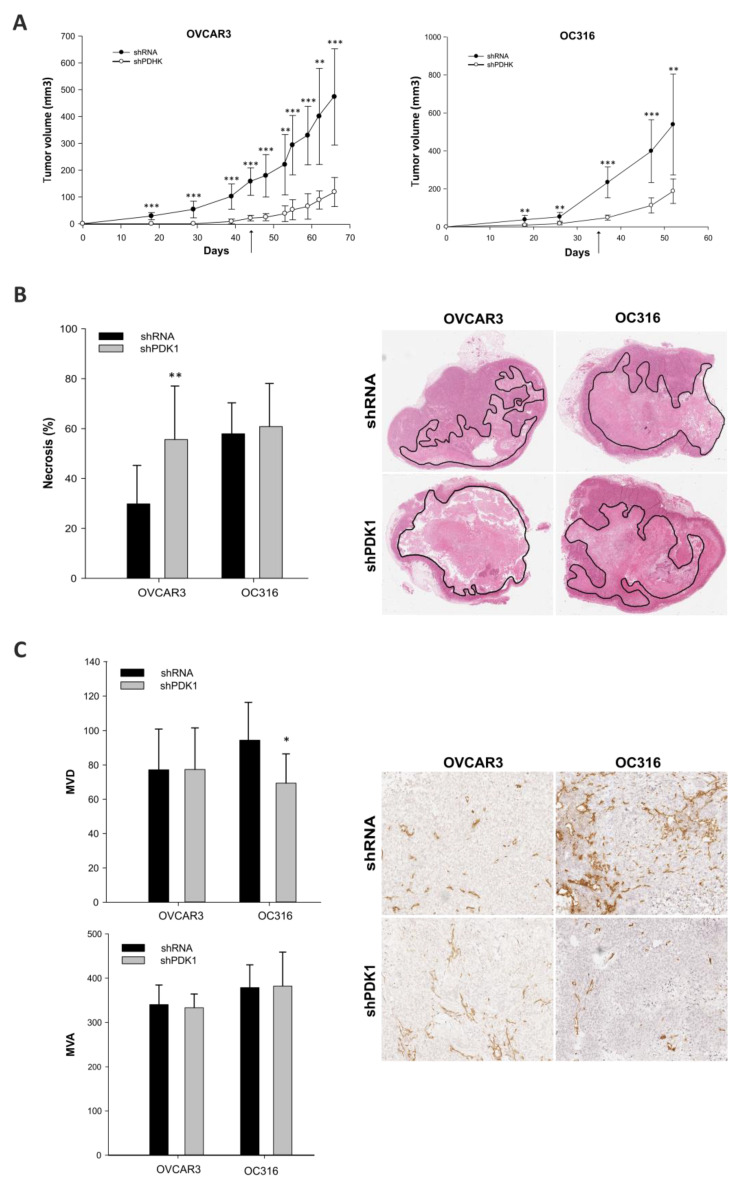
Effects of PDK1 silencing on tumor growth and angiogenesis. (**A**) Kinetics of tumor development in NOD/SCID mice s.c. injected with silenced cells (shPDK1) and control cells (shRNA) in OVCAR3, and OC316, (n = 4 mice for group), ** *p* < 0.01 *** *p* < 0.001, Mann–Whitney test. Arrows indicate the time of sacrifice in control tumors harvested when their volumes were comparable to shPDK1 tumors—i.e., 100 and 200 mm^3^ in the case of OVCAR3 and OC316 tumors, respectively. (**B**) Histologic images and columns indicate quantitative analysis of necrotic areas in n = 8 different tumors for each group, ** *p* < 0.01, Mann–Whitney test. Original magnification 1.25×. (**C**) Microvessel density (MVD) and microvessel area (MVA) evaluations were quantified by image analysis software on digitalized slides. * *p* < 0.05. Columns show mean ± SD values (n = 8 fields for tumor; n = 4 tumors for group), *** *p* < 0.001, Mann–Whitney test. Original magnification 10×.

**Figure 5:**
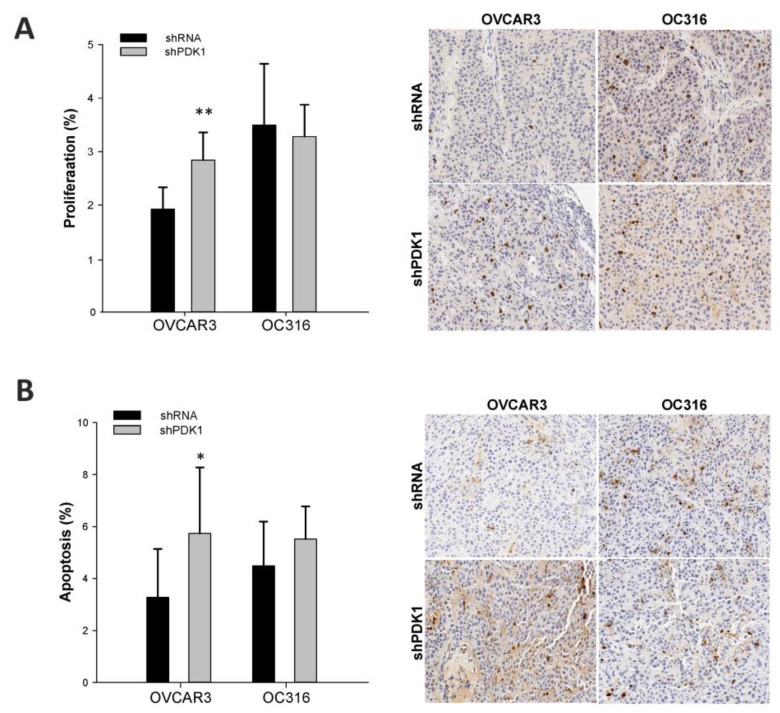
Effects of PDK1 silencing on tumor proliferation and apoptosis. (**A**,**B**) Proliferation and apoptosis of tumors were evaluated by immunohistochemical (IHC) expression of phospho-Histone H3 (pHH3) and caspase 3, respectively. Results were quantified by image analysis software on digitalized slides. Columns show mean ± SD values (n = 8 fields for tumor; n = 4 tumors for group), * *p* < 0.05, ** *p* < 0.01, Mann–Whitney test. Original magnification 20×.

Next, we performed an in vivo experiment to investigate the effects of PDK1 modulation on tumor growth and metabolism: shPDK1 or shRNA tumor cells from OVCAR and OC316 cell lines were injected subcutaneously in SCID mice (n = 4 mice/group). In both cases, growth of shPDK1-deficient tumors was markedly delayed compared to shRNA cells (Figure 4A). At tumor harvest, we found by qPCR that PDK1 mRNA expression levels were still reduced in shPDK1 compared to control tumors (Supplementary Figure S1), indicating that gene silencing effects were maintained. Since tumor necrosis tends to increase with tumor volume in s.c. tumor models, we evaluated this feature and found a positive association between volume and percentage of necrosis in OVCAR-3 and OC316 tumors (Supplementary Figure S2). Based on this finding, to prevent any bias due to tumor volume, all morphologic parameters were assessed in control tumors harvested when volume was comparable to that of shPDK tumors—i.e., 100 and 200 mm^3^ in the case of OVCAR3 and OC316 tumors, respectively. IHC analysis showed that in the OVCAR3 model shPDK tumors had more necrotic areas compared to shRNA tumors (Figure 4B), whereas in the case of OC316 tumors necrosis was substantial (>60% of the tumor area) but comparable between the two experimental groups (Figure 4B). We subsequently analyzed tumor angiogenesis and found reduced MVD in shPDK tumors compared to control OC316 tumors, whereas MVD values were similar in OVCAR3 tumors. Finally, mean vessel area was comparable in shPDK and control tumors in both tumor models (Figure 4C). Proliferation and apoptosis were evaluated by IHC staining with the pHH3 and activated caspase-3 markers, respectively. Results, shown in Figure 5, indicate that apoptosis and proliferation were increased in shPDK OVCAR3 tumors compared with controls, whereas in the case of OC316, tumor proliferation and apoptosis were comparable between the two experimental groups (Figure 5). All these morphologic parameters were quantified on the entire viable tumor area by using an innovative digital pathology approach, as shown in representative pictures in Supplementary Figure S3 and explained in the Materials and Methods section.

We conclude that PDK silencing is maintained in vivo and is associated with reduced growth of s.c. tumor xenografts. However, mechanisms underlying these tumor growth delaying effects appear to be heterogeneous in the two tumor models investigated. In fact, in the case of OC316 tumors, the only parameter correlated with reduced tumors growth is MVD, hinting at a primary role of angiogenesis. In contrast, in the OVCAR3 tumor model PDK silencing is associated with increased necrosis, apoptosis and cell proliferation without significant differences in angiogenesis.

### 3.3. Gene Expression Profiles of PDK-Silenced Tumors

**Figure 6:**
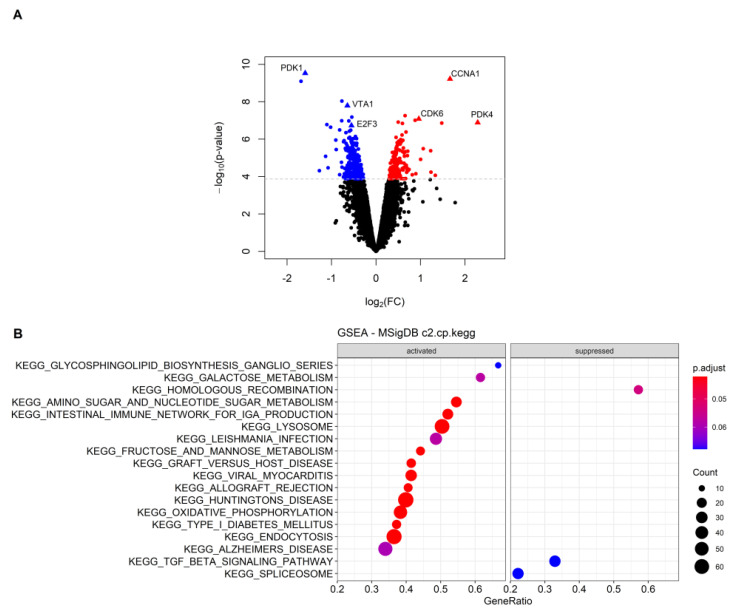
Differentially expressed genes and KEGG pathways significantly enriched in shPDK1 vs. control OVCAR3 tumors. (**A**) Volcano plot showing significantly upregulated (red) and downregulated (blue) genes in the comparison shPDK1 vs. shRNA OVCAR3 tumors. The x-axis represents the log2(FC) and the y-axis the −log10 (*p*-value). Dashed grey line corresponds to the cut-off of 0.01 on Benjamini–Hochberg adjusted *p*-value used to determine DEGs. Interesting genes explicitly cited within the manuscript are indicated with triangles and annotated. (**B**) Dot plot showing the results of Gene Set Enrichment Analysis (GSEA) on the KEGG pathway from “c2.cp.kegg” subcollection of the MSigDB. The 15 most significantly activated and the 3 significantly suppressed pathways, considering a Benjamini–Hochberg (BH) adjusted *p*-value < 0.10, are displayed. The size of each dot (count) represents the number of genes contributing to the enrichment of that pathway, and the color represents the Benjamini–Hochberg adjusted *p*-value (red dots are the most significant). Enriched KEGG pathway are ordered by decreasing gene ratio (count/size of the gene set).

**Figure 7:**
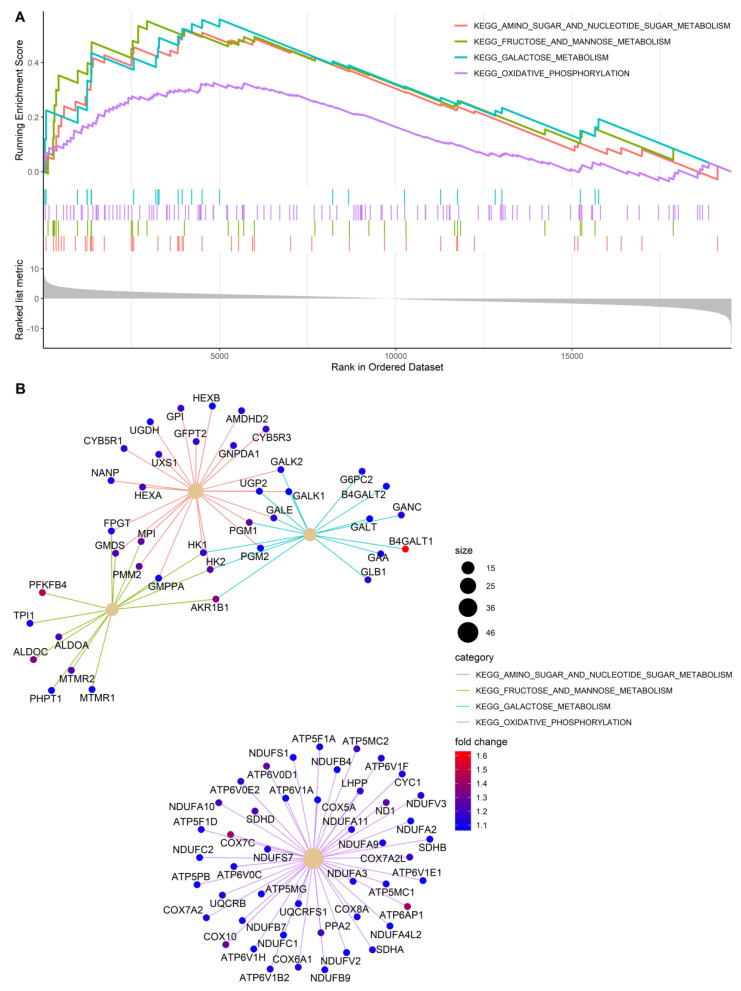
Metabolic pathways significantly activated following PDK1 silencing in OVCAR3 tumors by GSEA. (**A**) Enrichment plots of the four metabolism-related KEGG pathways significantly activated in tumors formed by OVCAR-3 cells bearing PDK1 silencing. GSEA results are visualized in terms of running enrichment scores and preranked lists. (**B**) Gene-concept network showing the significant genes contributing to the positive enrichment of the four metabolism-related KEGG pathways and their fold changes (color ramp on side). The size of the nodes representing the categories is proportional to the number of genes contributing to their enrichment. Interestingly, while the activation of oxidative phosphorylation pathway is determined by a distinct set of genes, the other three metabolic pathways (i.e., amino sugar and nucleotide sugar metabolism, fructose and mannose metabolism and galactose metabolism) appear strongly interconnected with several shared core genes.

To investigate at the transcriptional level the effects of PDK silencing in tumors, and correlate gene expression profiles with the morphologic features described above, we performed microarray analysis of OVCAR3 tumors. This model was selected based on the large number of morphological parameters (including necrosis, apoptosis and proliferation) distinguishing shPDK from control tumors. Bioinformatics analysis of transcriptomic data revealed 472 differentially expressed genes (with Benjamini–Hochberg adjusted *p*-value < 0.01), of which 163 were upregulated and 309 were downregulated in PDK1-silenced vs. control tumors. Despite a significant alteration, most of the genes showed mild modulation in terms of fold change, as demonstrated by volcano plot representation (Figure 6A). Interestingly, among the top downregulated genes in PDK1-silenced tumors was PDK1—in line with results of qPCR analysis shown above—whereas PDK4, another member of the PDK family, was strongly upregulated (Figure 6A). We also found increased levels of CDK6 and cyclin A1, fitting with results of pHH3 staining, and reduced vesicle trafficking and E2F transcription factor 3 expression (Supplementary Table S1 and Figure 6A). Some angiogenesis-related transcripts, including FGF2, PDGFA-B, were downregulated whereas others (CXCL-8) were upregulated in PDK1-silenced tumors (Supplementary Table S2). A Gene Set Enrichment Analysis (GSEA) was performed on the KEGG pathways in the MSigDB (c2.cp.kegg subcollection) to highlight any coordinated alteration of groups of genes. Several metabolism-related KEGG pathways were activated in PDK1-silenced tumors, including amino sugar and nucleotide sugar metabolism, fructose and mannose metabolism, galactose metabolism and oxidative phosphorylation, among others (Figure 6B). In contrast, only three KEGG pathways (i.e., homologous recombination, TGFβ signaling pathway and spliceosome) were significantly downregulated in PDK1-silenced compared with control tumors (Figure 6B). Gene set enrichment plots and a graphic representation of the genes contributing to the positive enrichment of the aforementioned metabolic pathways in PDK1-silenced tumors and their interconnections are presented in Figure 7A,B. Altogether, RNA profiling disclosed increased glucose catabolic pathways—comprising both oxidative phosphorylation (OXPHOS) and glycolysis—in PDK1-silenced tumors, in line with the high mitotic activity detected in the viable rim of these tumors.

## 4. Discussion

State-of-the-art knowledge connects high glycolytic activity with increased malignancy and both autonomous cancer cell mechanisms (proliferative activity) and tumor microenvironment mechanisms (angiogenesis and immunomodulatory effects) are likely to account for this association. However, the impact of forced attenuation of glycolysis on tumor growth, angiogenesis, and metabolism has been marginally explored. In this study, we investigated this issue in ovarian cancer xenografts by using genetic silencing of PDK, an established regulator of glycolysis at the center of significant basic and clinical research interests in the past decade as the main pharmacologic target of dichloroacetate (DCA) [15]. Previous studies, mainly focusing on pharmacologic inactivation of PDK, have illustrated how DCA shifts tumor metabolism from glycolysis to OXPHOS and the impressive negative effects of PDK blockade on growth of various types of experimental tumors (reviewed in [16]). In the context of ovarian cancer, a recent study investigated effects of PDK1 silencing on cell motility and resistance to cisplatin [27].

In our study, we focused on in vivo experiments, which were made possible by the persistence of PDK1 silencing in tumor cells, as shown by prolonged in vitro culture of genetically modified tumor cells as well as by analysis of PDK1 levels in tumors obtained >2 months after s.c. injection of tumor cells. Our results indicate that in vivo antitumor effects are much more prominent compared with the mild growth delaying effects associated with PDK1 silencing in vitro, suggesting the existence of indirect underlying mechanisms. Moreover, our findings hint at an impact of PDK1 silencing on angiogenesis but tumor model-specific differences appeared to be prominent. In the highly glycolytic OC316 model, necrosis was very abundant (>60%) and was apparently not further increased by PDK1 silencing. In this model, the only parameter significantly different between the two compared tumor groups was MVD. Thus, modulation of angiogenesis appears to be the prevalent mechanism of action downstream of PDK1 silencing in OC316 cells. In contrast, in the OVCAR-3 tumor model, no significant difference in MVD was found but the increased necrosis in PDK1-silenced tumors indirectly suggests that PDK1 silencing strongly impacted on formation of new vessels by making the vascular network insufficient to maintain tumor viability. Values measured around the necrotic area are reminiscent of viable tumor rims found in tumors treated with vascular-damaging agents (VDAs) [28]. In fact, VDAs cause rapid (within hours) vascular damage, hypoxia, and tumor necrosis followed by an inflammatory reaction which boosts tumor cell proliferation and angiogenesis. Similar features were observed in the viable tumor area of OVCAR-3 tumors. The fact that tumor size was significantly smaller than in controls can account for increased tumor necrosis and apoptosis, which are predominant features in this model and likely outweigh proliferation. These results are also in line with transcriptomic studies, which detect increased OXPHOS activity in PDK1-silenced OVCAR-3 tumors but also pinpoint increased sugar metabolism, which is a biochemical marker of cell proliferation. Along this line, the persistently low expression levels of PDK1 are likely compensated by increased PDK4 expression.

One limitation of our study regards the cancer cell lines used. In fact, while OVCAR3 is recognized as an almost certain model of high grade serous ovarian cancer [29,30], OC316 has an hypermutated genotype and uncertain histologic origin [31]. Speculatively, some intrinsic features of the cell lines used could account for differences observed after PDK1 silencing and our findings would certainly be strengthened by testing additional ovarian cancer cell line models.

Our data demonstrate that effects of PDK1 attenuation are much more complex in vivo than in vitro. In agreement with previous studies [32,33,34,35], PDK1 silencing lowered glycolysis and promoted OXPHOS in tumor cells grown in vitro; in the context of the tumor microenvironment, the scenario is rather different. Speculatively, the primary effect of PDK1 silencing on angiogenesis leads to a secondary effect of increased necrosis, which then triggers, as a tertiary effect, a complex tumor microenvironment response which substantially differs from the straight effects of PDK1 silencing detected in vitro. In agreement with this hypothesis, transcriptomic data indicate increased metabolic activity of PDK1-silenced tumors, as opposed to reduction in glycolysis and increased OXPHOS detected in vitro.

Altogether, our findings add new evidence in support of a link between tumor metabolism and angiogenesis and remark the importance of investigating net effects of modulations of metabolic pathways in the context of the tumor microenvironment.

## Data Availability

Microarray data generated during the study have been deposited in the ArrayExpress database (www.ebi.ac.uk/arrayexpress) under accession number E-MTAB-9877.

## Supplementary Materials

The following are available online at https://www.mdpi.com/2073-4409/10/2/325/s1. Figure S1. Decreased expression of PDK1 mRNA in tumors. Figure S2. Quantification of tumor necrosis in subcutaneous OVCAR-3 and OC316 tumor xenografts. Figure S3. Examples of markers analyzed by digital pathology. Table S1. Differentially expressed genes in shPDK1 tumors vs. controls. Table S2. List of angiogenesis-related transcripts and their modulation in PDK-silenced tumors.
