# Supplementary material for: Genetic Perturbation of Pyruvate Dehydrogenase Kinase 1 Modulates Growth, Angiogenesis and Metabolic Pathways in Ovarian Cancer Xenografts
Source: Cells. 2021 Feb 5;10(2):325. doi: 10.3390/cells10020325 (PMC7915933; doi:10.3390/cells10020325)
Supplement: Supplementary file 1 [file cells-10-00325-s001.zip › cells-1048808-SI-layout/Supplementary files/Supplementary Figures - paper PDK1 v6_REVISED.docx]

**
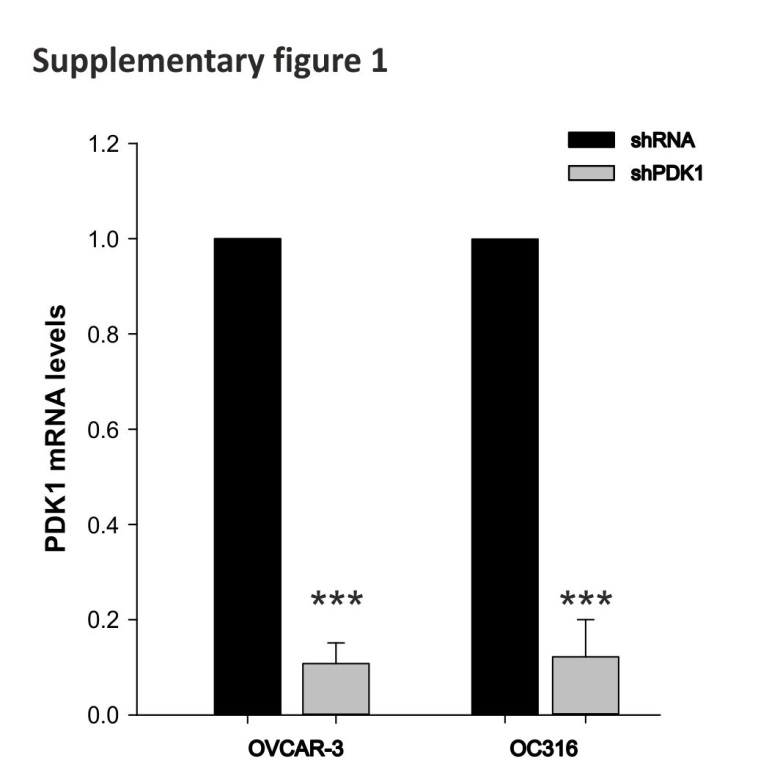
**

**Supplementary figure 1. Decreased expression of PDK1 mRNA in tumors.** OVCAR-3 and OC316 tumors (n=6 per group) were analyzed at sacrifice. PDK1 mRNA expression levels were analyzed by real-time PCR. ***p < 0.01 Mann-Whitney.


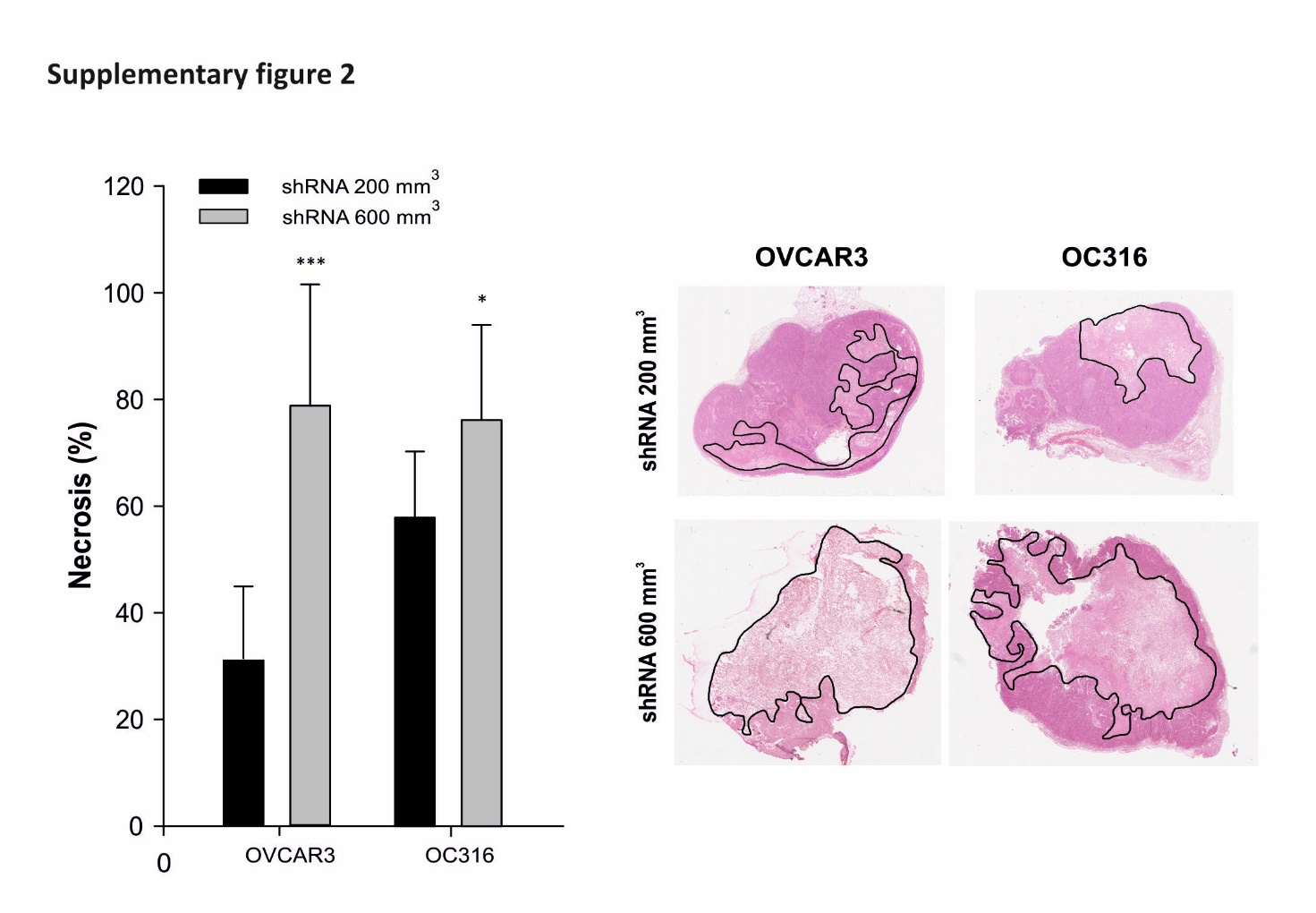


**Supplementary figure 2. Quantification of tumor necrosis in subcutaneous OVCAR-3 and OC316 tumor xenografts.** Presence of necrosis was compared between tumors formed by control ovarian cancer cells (with shRNA). Results show association between tumor volume and the percentage of necrotic areas. Columns show mean ± SD values (*n* = 8 fields for tumor; *n* = 4 tumors for group), * p < 0.05 ***, *p* < 0.001, Mann Whitney test.

**
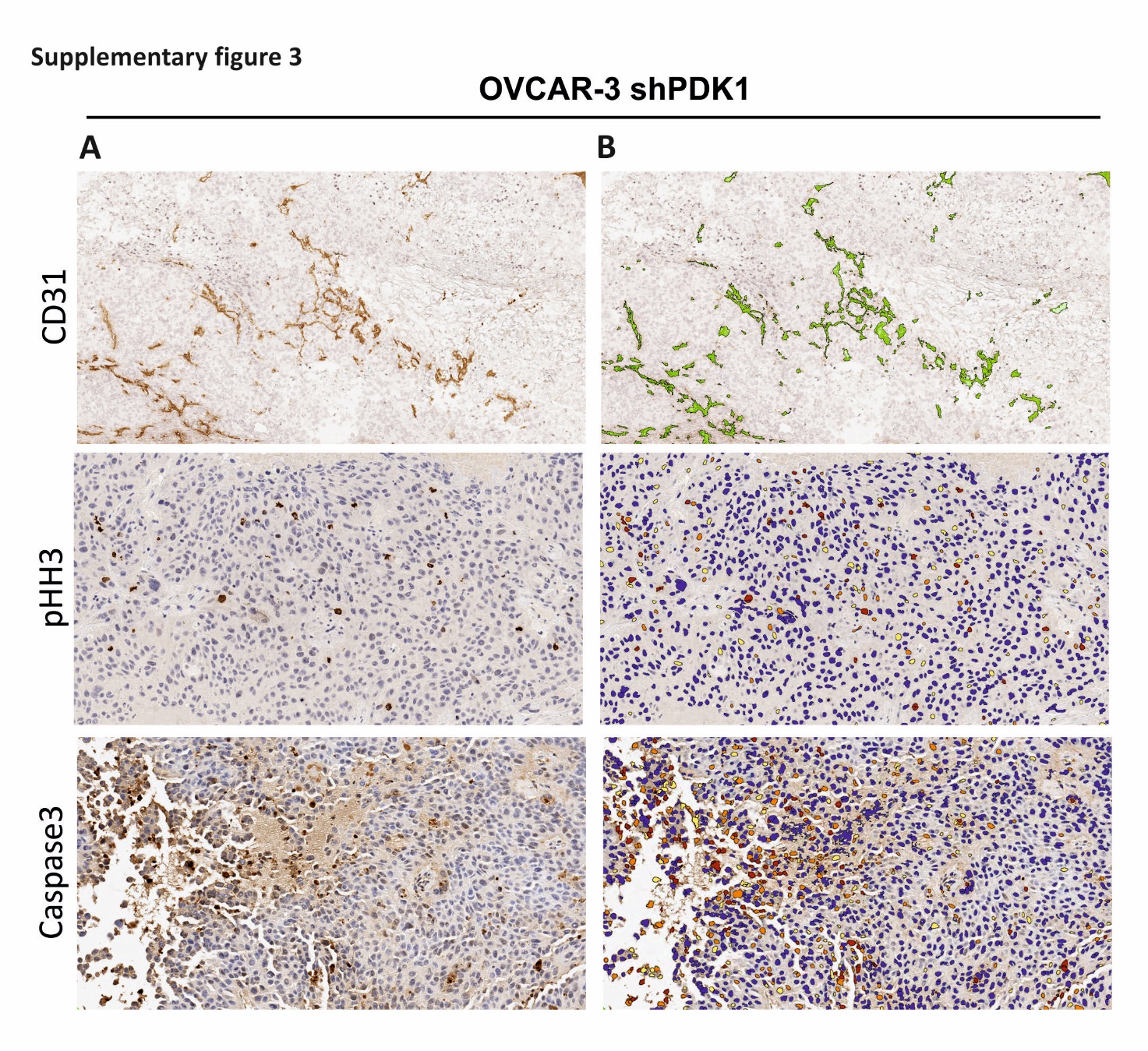
**

**Supplementary figure 3. Examples of markers analysed by Image Scope software. (A)** Immunohistochemical staining of CD31, pHH3, Caspase3 in representative OVCAR-3 tumors silenced for PDK1. **(B)** Same slides with visualization of mark-up of respective algorithms. The macro used for the analysis of CD31 was Aperio microvessels (vessels showed in green). Algorithm used for pHH3 and Caspase3 was Aperio nuclear macro (positive nuclei are showed in red), that classifies nuclei as positive (3+/red) (B) or as negative (0+/yellow) (D). Original magnification 10X for CD31 slides and 20x for pHH3 and Caspase 3.

**Supplementary Table 1. Differentially expressed genes between shPDK1 tumors vs. controls with BH adjusted p-value<0.01.** The table is provided as a separated Excel file.

**Supplementary Table 2.** **List of angiogenic factor genes differentially expressed between shPDK1 OVCAR3 tumors and controls**

| **PROBE ID** | **SYMBOL** | **GENE NAME** | **FC** | **p-value** | **BH adjusted p-value** |
| --- | --- | --- | --- | --- | --- |
| 11729062_a_at | PDGFA | platelet derived growth factor subunit A | -1.290 | 7.22E-05 | 0.0075 |
| 11731665_a_at | PDGFB | platelet derived growth factor subunit B | -1.217 | 7.81E-04 | 0.0263 |
| 11731667_s_at | PDGFB | platelet derived growth factor subunit B | -1.256 | 1.47E-03 | 0.0367 |
| 11725040_at | FGF2 | fibroblast growth factor 2 | -1.383 | 2.58E-03 | 0.0501 |
| 11725041_at | FGF2 | fibroblast growth factor 2 | -1.191 | 6.46E-03 | 0.0828 |
| 11754026_a_at | CXCL8 | C-X-C motif chemokine ligand 8 | 1.552 | 7.79E-03 | 0.0921 |

Fold change (FC) values refer to the comparison of shPDK1 OVCAR3 tumors vs. control. Angiogenic factor genes that appeared at least weakly significant, with Benjamini-Hochberg (BH) adjusted p-value < 0.10, were reported in the table. PDGFA, PDGFB, FGF2 and CXCL8 resulted differentially expressed. Differently, VEGFA, VEGFB, VEGFC, VEGFD and PGF appeared among the non-significant angiogenic factor.
